# Unlike Many Disease Resistances, Rx1-Mediated Immunity to Potato Virus X Is Not Compromised at Elevated Temperatures

**DOI:** 10.3389/fgene.2020.00417

**Published:** 2020-04-24

**Authors:** Manon M. S. Richard, Marijn Knip, Thomas Aalders, Machiel S. Beijaert, Frank L. W. Takken

**Affiliations:** Swammerdam Institute for Life Sciences (SILS), Molecular Plant Pathology, University of Amsterdam, Amsterdam, Netherlands

**Keywords:** sensor NLR, helper NLR, plant immunity, temperature, virus, thermotolerance, disease triangle

## Abstract

Specificity in the plant immune system is mediated by Resistance (R) proteins. Most R genes encode intracellular NLR-type immune receptors and these pathogen sensors require helper NLRs to activate immune signaling upon pathogen perception. Resistance conferred by many R genes is temperature sensitive and compromised above 28°C. Many Solanaceae R genes, including the potato NLR Rx1 conferring resistance to Potato Virus X (PVX), have been reported to be temperature labile. Rx1 activity, like many Solanaceae NLRs, depends on helper-NLRs called NRC’s. In this study, we investigated Rx1 resistance at elevated temperatures in potato and in *Nicotiana benthamiana* plants stably expressing *Rx1* upon rub-inoculation with GFP-expressing PVX particles. In parallel, we used susceptible plants as a control to assess infectiousness of PVX at a range of different temperatures. Surprisingly, we found that Rx1 confers virus resistance in *N. benthamiana* up to 32°C, a temperature at which the PVX::GFP lost infectiousness. Furthermore, at 34°C, an Rx1-mediated hypersensitive response could still be triggered in *N. benthamiana* upon PVX Coat-Protein overexpression. As the Rx1-immune signaling pathway is not temperature compromised, this implies that at least one *N. benthamiana* helper NRC and its downstream signaling components are temperature tolerant. This finding suggests that the temperature sensitivity for Solanaceous resistances is likely attributable to the sensor NLR and not to its downstream signaling components.

## Introduction

Plants have developed a multi-layered immune system activated by receptor proteins that detect pathogen-generated molecules. Immune receptors can be classified into two main groups: (i) the extracellular receptors, mainly Receptor like-Kinases (RLK) or -Proteins (RLP), commonly associated with either recognition of pathogens’ conserved features (microbial- or pathogen associated molecular patterns, MAMP or PAMP) or by pathogen inflicted damage (damage associated molecular patterns or DAMP) and (ii) Intracellular receptors. Members of this latter group often encode Nucleotide-binding domain and leucine-rich repeat (NLR) proteins that recognize specific pathogen encoded avirulence factors (Avr) (Dodds and Rathjen, 2010). NLRs can be divided into two major sub-groups according to their N-terminal domain, the TNLs, with a Toll/interleukin-1 receptor (TIR) and the CNLs, with a Coiled Coil (CC) domain (Monteiro and Nishimura, 2018). NLRs have been described as molecular switches that turn ON immune signaling after pathogen perception (Takken et al., 2006). NLR activation often triggers local cell death, the so-called hypersensitive response (HR) (Balint-Kurti, 2019).

In plant genomes, NLRs are typically encoded by a large gene family consisting of several hundreds of genes. NLRs can be categorized into two operative groups, the sensors (e.g., NLRs responsible for pathogen perception) and the transducers (or helpers). The latter group has recently been highlighted and is responsible for relaying or translating the upstream signal from the sensor NLR to the downstream signaling components (Wu et al., 2018). In Solanaceae, a phylogenetically related NLR family, consisting of NLR Required for Cell death (NRC) genes, have been described as helper NLRs (Adachi et al., 2019). Required by a large number of sensor NLRs that mediate resistance against diverse pathogens, they constitute a complex network of immune receptors (Wu et al., 2017). For example, the sensor NLRs Mi-1 from tomato, Rpi-blb2 and R1 from potato rely exclusively on NRC4 to trigger resistance responses, while the tomato NLR Prf and the potato NLR GPA2 can trigger HR via NRC2 or NRC3. Other NLRs such as Rx1 from potato or Bs2 from pepper can pair with either NRC2, NRC3, or NRC4 (Wu et al., 2017). Interestingly, the founder NRC, NRC1, has been initially identified to be required for resistance mediated by the non-NLR, Cf-4 that confers resistance toward the fungus *Cladosporium fulvum* in tomato (Gabriels et al., 2007). This finding suggests a potential role of these helpers to integrate immune signaling from both intra- and extracellular immune receptors (Leibman-Markus et al., 2018).

Although environmental conditions, such as temperature, have a crucial impact on the outcome of the diseases, this third component of the disease triangle (plant, pathogen, and environment) is often overlooked in plant-pathogen interaction studies. A temperature dependency of disease resistance has been reported in several cases involving different kind of pathogens, such as viruses, fungi, oomycetes, bacteria, or nematodes. For example, the tobacco NLR N is unable to confer resistance to the Tobacco mosaic virus (TMV) above 28°C (Whitham et al., 1996). Resistance to the nematode *Meloidogyne incognita* mediated by the NLR Mi-1 in tomato is compromised by exposure to 35°C for 3 h preceding inoculation (De Carvalho et al., 2015). *Tsw-*mediated resistance fails to trigger resistance to Tomato spotted wilt virus (TSWV) at 32°C and above in pepper plants (Moury et al., 1998). The NLR Bs2 from pepper, conferring resistance to the bacterium *Xanthomonas axonopodis* pv. *vesicatoria*, shows compromised resistance and HR at 32°C (Romero et al., 2002). Interestingly, non-NLR mediated resistance; such as resistance mediated by the transmembrane receptor like proteins Cf-4 and Cf-9 against *C. fulvum* is also impaired at elevated temperature (Cai et al., 2001; De Jong et al., 2002).

While temperature sensitivity of resistance seems widespread, it is not trivial to study this aspect in many plant pathogen interactions. One reason is that pathogen fitness and virulence can also be affected by (elevated) temperatures (Velasquez et al., 2018), complicating identification of temperature sensitive components in an interaction. Therefore, as a proxy for immune activation at elevated temperatures, the capacity of R genes to trigger HR upon (over)expression of their corresponding *Avr* is often used. For example, in Wang et al. (2009) N- and Rx1-temperature sensitivity is assessed by monitoring loss of HR when co-expressed with the corresponding *Avrs*; *p50* from TMV and the *Coat Protein* (CP) from Potato mosaic virus (PVX) at 28 and 30°C, respectively (Wang et al., 2009). However, HR is not always correlated with functional resistance. For example, HR triggered by the recognition of *Pseudomonas syringae* pv. *tomato* DC 3000 (PtoDC3000) HopZ1a or AvrRpt2 in *Arabidopsis thaliana* is suppressed at 30°C, while resistance to the bacteria is unaffected (Menna et al., 2015). Additionally, co-expression of *R* and or *Avr* gene(s) in heterologous systems often relies on *Agrobacterium*-mediated transient transformation assays (ATTA). A drawback of this system is the temperature sensitivity of T-DNA transfer by *Agrobacterium tumefaciens*, which makes plant transformation above 27°C highly inefficient (Dillen et al., 1997).

Many Solanaceae resistances, mediated by NLR- or non-NLR sensors that depend on NRC helpers, are reported to be compromised at elevated temperature. However, the temperature sensitive component of their molecular signaling pathways (sensor, helper, or downstream signaling) remains unknown. Since this temperature sensitivity concerns resistance triggered by different types of receptors (NLR and non-NLR, such as the RLPs Cf-4 and Cf-9), it is tempting to speculate that shared downstream signaling components, such as the helpers NRCs, could be the Achilles’ heel of the immune signaling at elevated temperatures. Rx1 is a well-studied NLR from potato and is a perfect model to challenge our hypothesis since it can signal via NRC2, NRC3, or NRC4 (Wu et al., 2017).

The NLR Rx1 triggers resistance to PVX upon recognition of its CP in potato and in *N. benthamiana* stably expressing Rx1 from its native promoter (Bendahmane et al., 1999). Rx1 confers a so called “extreme resistance” response that prevents viral replication without triggering cell death (Bendahmane et al., 1999). Overexpression of the avirulent *CP* (*CP106*) in an *Rx1*-expressing plant nonetheless can trigger HR in heterologous species such as *N. benthamiana*, whereas CP105, a CP variant of an Rx1 resistance breaking strain of PVX, does not (Goulden et al., 1993; Bendahmane et al., 1999). Rx1 has been reported to be temperature sensitive as it was unable to trigger HR at 30°C upon ATTA-mediated *CP*-expression in *N. benthamiana* (Wang et al., 2009). However, the capacity of Rx1 to mount resistance against PVX at elevated temperature is not known.

Here we investigate Rx1-mediated resistance to PVX in *N. benthamiana* plants stably expressing Rx1 from its native promoter and in potato. Infection with the GFP-expressing PVX particles (PVX::GFP) was done by rub inoculation and is independent of *Agrobacterium* transformation. We observed that, in *N. benthamiana*, Rx1 is preventing PVX::GFP replication up to temperatures above which PVX::GFP is no longer infectious. The temperature resilience of Rx1 in potato could not be assessed as the virus itself is not efficiently infecting potato at 30°C in our experimental set-up. Besides, we assessed the capacity of Rx1 to trigger HR at elevated temperatures using *Rx1 N. benthamiana* plants stably transformed with a dexamethasone (DEX)-inducible *CP* construct. We observed that Rx1-mediated HR can still be observed at temperatures up to 34°C. Altogether, our results imply that Rx1 is a thermotolerant R protein – as are its downstream signaling components – providing new insights in the mechanisms underlying thermosensitivity in plant immunity.

## Materials and Methods

### Plant Lines, *N. benthamiana* Transformation and Growing Conditions

Wild-type and transgenic *Rx1:4xHA* (Lu et al., 2003), expressing Rx1 under control of its native promoter, *N. benthamiana* plants were used for PVX bioassays. To monitor HR *Rx1:4xHA+DEX::CP106 9-4* (referred to as *Rx1D106*, internal identifier #FP1807) and *Rx1:4xHA+DEX::CP105 6-6* (referred to as *Rx1D105*, internal identifier #FP1810) stable transgenic lines were generated. For this, *N. benthamiana Rx1:4xHA* plants were transformed using the dexamethasone (DEX) inducible PVX-CP constructs, either *DEX::CP106 or DEX::CP105* described in Knip et al. (2019), using *Agrobacterium-*mediated transformation as described in Sparkes et al. (2006). Briefly, *A. tumefaciens* infiltrated leaves were surface sterilized, cut into 2 cm^2^ diamond shape pieces, and placed on shoot-induction medium supplemented with 14.8 μg/ml hygromycin for selection. Shoots from putative transformants were transferred to root-induction medium containing 14.8 μg/ml hygromycin. Ten and seven candidate transformants for *DEX::CP106* or *DEX::CP105* constructs, respectively, were selected for seed production. Segregation for hygromycin resistance of the obtained T1 progeny was assessed on selective medium and seven and five lines with a single insertion were identified for *DEX::CP106 or DEX::CP105* constructs, respectively. Homozygosity of T = 2 plants was evaluated by Real-Time PCR using gDNA by estimation of t-DNA copy number (by amplification of the Hygromycin resistance gene, using the oligonucleotides FP7722-HP ThygroFW: GTTCGGGGATTCCCAATACGAGGTC and FP7723-HPThygroRV: ATCGAAATTGCCGTCAACCAAGCTC) compared to a endogenous reference gene (NRG1, amplified using the oligonucleotides FP8254: GTGTCCGACCACTAAGCATGGAACTA and FP8255: CTGCTGGTGCATCCTTTCTGGAAATC). The Real-Time PCRs were performed in QuantStudioTM3 (Thermo Fisher Scientific). The 10 μL PCR contained 0.2 μM of each primer, 100 ng of gDNA, 0.05 μl of DNA polymerase (DreamTaq, Thermo Fisher Scientific), 1x Evagreen dye (Solis Biodyne), 1x ROX passive reference, dNTPs, and water. The cycling program was set to initial denaturation 2 min at 94°C, 40 cycles; denaturation for 15 s at 95°C, annealing for 20 s at 58°C, elongation for 30 s at 72°C, followed a melting curve analysis of 15 s at 95°C, 1 min at 60°C, 15 s at 95°C. The copy number analysis was performed using the online Thermo Fisher Scientific application. Plants being phenotypically similar to the parental *Rx1:4xHA* plants, and showing expression of the *CP* upon dexamethasone treatment were selected, resulting in three and two independent homozygous *Rx1D106* and *Rx1D105* lines, respectively. One out of the three *Rx1D106* and one out of the two *Rx1D105*, were used for this study as the different *Rx1D106* and *Rx1D105* lines showed identical responses when DEX treated (data not shown).

*N. benthamiana* plants were grown under long-day conditions in a climate chamber (22°C, 70% humidity, 16 h/8 h light/dark) for 3–4 weeks. Potato tubers from two diploid potato genotypes RH 89-039-16 (RH) and SH82-93-488 (SH) (Van der Voort et al., 1997) were planted in soil and plants were grown for 6 weeks under the conditions described above. One day before treatment (either PVX::GFP rub-inoculation or dexamethasone treatment, see below), plants were transferred and incubated in a MD1400 MODULAR CLIMATE CHAMBER (Snijders Labs) at the indicated temperatures under a constant humidity of 80% at a 12/12h light/dark regime.

### PVX::GFP Rub-Inoculation and *in planta* Virus Detection in *N. benthamiana*

To produce infectious PVX::GFP particles, leaves of 4 weeks old WT *N. benthamiana* plants were agroinfiltrated with an *A. tumefaciens* GV3101 strain containing the *pJIC SA_Rep* helper plasmid and the *PVX::GFP* construct (internal identifier BglFP#4081) according to Ma et al. (2012). *PVX::GFP* was obtained by inserting the *LSS-msfGFP* ORF^1^ after a duplicated CP promoter, into SgsI – NotI restriction sites of pGR106, a binary vector containing an infectious PVX clone (Jones et al., 1999). Two weeks after agroinfiltration, systemically infected leaves were either snap frozen with liquid nitrogen and stored at –80°C or directly used for rub-inoculation. PVX::GFP inoculum was made by grinding a fresh, or frozen, PVX::GFP infected leaf with a mortar in 4 mL 50 mM potassium phosphate buffer pH 7. The youngest fully expanded leaves of 3 weeks old *N. benthamiana* plants (WT or *Rx1*) were mechanically inoculated by rubbing the adaxial side with a piece of miracloth (Merck; pore size of 22–25 μm) soaked in the PVX::GFP inoculum using Carborundum as an abrasive. Five minutes post-inoculation the inoculated leaves were rinsed with tap water and excess water was removed using paper tissues. Ten days after rub-inoculation, plants were photographed using a Panasonic Lumix DMC-LX15 camera placed in a dark chamber (Extraneous Light Protector and RS 1 stand, Kaiser, Germany) illuminated with UV light (RB 5003 UV Lighting Unit code n°5591, Kaiser, Germany).

### RNA Isolation and RT-PCR

For PVX::GFP RNA detection, systemic leaves from PVX::GFP rub-inoculated plants were sampled 10 days post-inoculation. To verify induction of PVX-CP transcription after dexamethasone treatment, leaves of *Rx1D105* and *Rx1D106* were sampled at 0, 2, and 4 h post-dexamethasone induction (hpdi). Total RNA was extracted using TRIzol LS reagent (Thermo Fisher Scientific, Waltham, MA, United States). The RNA was treated with DNase (Thermo Fischer Scientific) according to the supplier’s protocol and RNA concentrations were determined by measuring the absorbance at 260 nm on a NanoDrop (Thermo Fisher Scientific). cDNA was synthesized from 1.5 μg of total RNA using RevertAid H reverse transcriptase and Oligo-dT (Eurofins) in the presence of the RNAse inhibitor Ribolock (Thermo Fisher Scientific) following the supplier’s protocol and diluted 5 times in Milli-Q H_2_O.

Semi quantitative Reverse-Transcriptase (RT) PCR (35 cycles, annealing temperature of 60°C) was performed on 1 μl of diluted cDNA using DreamTaq DNA Polymerase (Thermo Fisher Scientific) following the supplier’s protocol, using CP specific primers FP8371-PVX-CP-F: CACTGCAGGCGCAACTCC and FP8372-PVX-CP-R: GTCGTTGGATTGYGCCCT or EF1α primers FP8391-NbEF1α-F: AGCTTTACCTCCCAAGTCATC and FP8392-NbEF1α-R: AGAACGCCTGTCAATCTTGG as a positive internal control.

### Potato Inoculation and PVX Detection by ELISA and Western Blot

Six terminal leaflets from 6 weeks old potato plants SH and RH genotypes were rub-inoculated with PVX::GFP as described above. Plants were kept at either 20 or 30°C prior to analysis. One week after inoculation, a quarter of each inoculated leaflet was sampled and homogenized in 50 mM Sodium Phosphate buffer pH 7, using a Tissuelyser (QIAGEN) and three 3 mm steel beads at 30 Hz for twice 30 s. The virus concentration was determined by DAS-ELISA using PVX antibodies (Prime Diagnostics, Wageningen, The Netherlands). Plates (NUNC-Immuno Plates Maxisorp F96) were coated with a 1:1000 dilution of the PVX polyclonal antibody to bind the antigen. A second polyclonal PVX antibody, conjugated with alkaline phosphatase, was used for detection by monitoring the conversion of the p-nitrophenyl phosphate substrate. Absorbance of each well was recorded at 405 nm with a reference filter of 655 nm using a BioTek Synergy H1 Hybrid multi-mode microplate reader (BioTek).

Proteins were isolated from one centimeter of petiole of each inoculated leaf 1 week post-inoculation using the method described in Knip et al. (2019). PVX detection by Western blot was performed on these samples as described in Knip et al. (2019) using PVX-specific antibody (diluted 1 : 3000) (ref 110411, Bioreba, Reinach, Switzerland).

### Rx1-Mediated HR Induction

One day after transfer of 3 weeks old *Rx1D106* and *Rx1D105* plants into the growth incubator at 20, 30, 32, 33, or 34°C, leaves were treated with 20 μM dexamethasone, 0.01% Silwet R-77 in Milli-Q H_2_O. The dexamethasone solution was applied on an ∼1 cm-diameter circle on the left side of each leaf. HR was assessed and pictures were taken 24 h post-dexamethasone application.

## Results

### Virulence of PVX Is Compromised Above 32°C While Rx1-Mediated Resistance Is Unaffected at This Temperature

**FIGURE 1:**
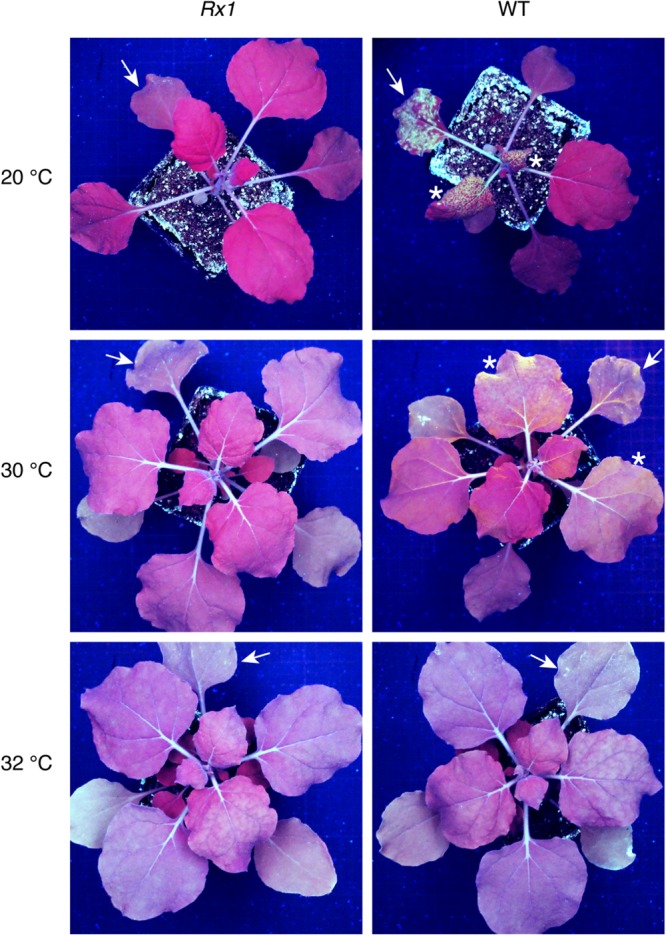
PVX::GFP fluorescence is observed in WT *N. benthamiana* plants up to 30°C but not in *Rx1* plants at the indicated temperatures. Detection of green PVX::GFP fluorescence under UV light in *Rx1* and WT *N. benthamiana* 10 days post-rub-inoculation. Arrows mark the rub-inoculated leaves and asterisks indicate systemic leaves emitting green fluorescence due to GFP accumulation.

**FIGURE 2:**
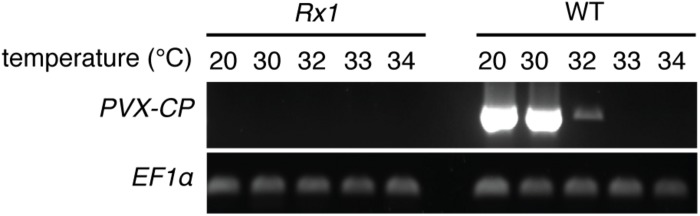
*PVX-CP* transcripts are detected in WT *N. benthamiana* plants up to 32°C, but not in inoculated *Rx1* plants. RT-PCR mediated detection of PVX::GFP RNA in systemic leaves of Rx1 and WT *N. benthamiana* 10 days post-rub-inoculation. Specific amplification of *PVX-CP* transcripts and plant *EF1α* transcripts are presented in the top -and bottom-row, respectively.

To determine a potential thermotolerance of Rx1-mediated resistance to PVX, *Rx1* transgenic *N. benthamiana* plants were rub-inoculated with infectious PVX particles at 20, 30, 32, 33, or 34°C. To visualize infection and spread of the virus, a recombinant PVX::GFP strain was used that triggers production of green fluorescent protein in infected plant cells. As a positive control for infection, susceptible wildtype (WT) *N. benthamiana* were rub-inoculated with the virus and incubated at the indicated temperatures. Ten days after inoculation WT plants kept at 20°C displayed strong PVX symptoms (leaf deformations and stunting) that correlated with intense GFP fluorescence under UV light (Figure 1). Green fluorescence could be observed at infection foci on the inoculated leaf and its petioles, and around the vasculature of systemically infected leaves (Figure 1). As expected, no symptoms nor green fluorescence were observed in PVX::GFP-inoculated *Rx1* plants at 20°C due to the resistance conferred by Rx1 (Figure 1). Instead, *Rx1* plants appeared red under UV light due to chlorophyll autofluorescence. RT-PCR on systemic leaf material confirmed that viral transcripts were present in WT plants, but absent in *Rx1* plants at 20°C (Figure 2). At 30°C and above, visual PVX symptoms could no longer be discerned in WT plants. However, GFP fluorescence could still be observed in systemic leaves of WT plants at 30°C, attesting the usefulness of a GFP reporter virus (Figure 1). In comparison, no GFP fluorescence was observed in *Rx1* plants inoculated at 30°C (Figure 1). These differences were confirmed by semi quantitative RT-PCR revealing the presence of viral RNA in the WT plant, but not in the resistant *Rx1* line (Figure 2). At 32°C, and above (data not shown), in addition to an absence of PVX symptoms, no GFP fluorescence was observed in WT or *Rx1* plants (Figure 1). Plants inoculated at 33 and 34°C are phenotypically similar to those incubated at 32°C (data not shown). Only a very small amount of viral RNA could be detected in the systemic leaves of WT plants at 32°C, but no viral transcripts were observed at 33 and 34°C (Figure 2). These findings suggests that at temperatures above 32°C PVX::GFP is no longer able to infect and spread in *N. benthamiana*. In addition, as no viral RNAs were detected at 32°C or above in *Rx1* plants, this suggests that *Rx1* is able to confer resistance to PVX::GFP in *N. benthamiana* at least up to temperatures at which the virus is no longer infectious.

The thermotolerance of Rx1-mediated resistance to PVX::GFP was investigated in potato plants with or without Rx1 resistance, the genotypes SH (Rx1) and RH (no Rx1), respectively. As expected, no virus was detected in the resistant SH genotype inoculated leaves or petioles at either 20 or 30°C (Supplementary Figures 1, 2). Notably, while PVX::GFP was detectable in sensitive RH potato inoculated leaves at 20°C, no virus could be detected in their petioles suggesting that the virus did not move systemically yet (Supplementary Figures 1, 2). Furthermore, no virus could be detected in susceptible RH inoculated leaves nor petioles at 30°C (Supplementary Figures 1, 2). This suggests that the capacity of PVX::GFP to infect at elevated temperature is determined by the host, as in *N. benthamiana* plants present in the same compartment high viral titers could be detected at 30°C (Supplementary Figures 1, 2). The inability of PVX::GFP to infect potato plants at elevated temperature in our experimental set-up prevents further investigation of the thermotolerance of Rx1 resistance in potato.

### Rx1 Triggers HR Upon Recognition of the Avirulent PVX Coat Protein Variant at 34°C

**FIGURE 3:**
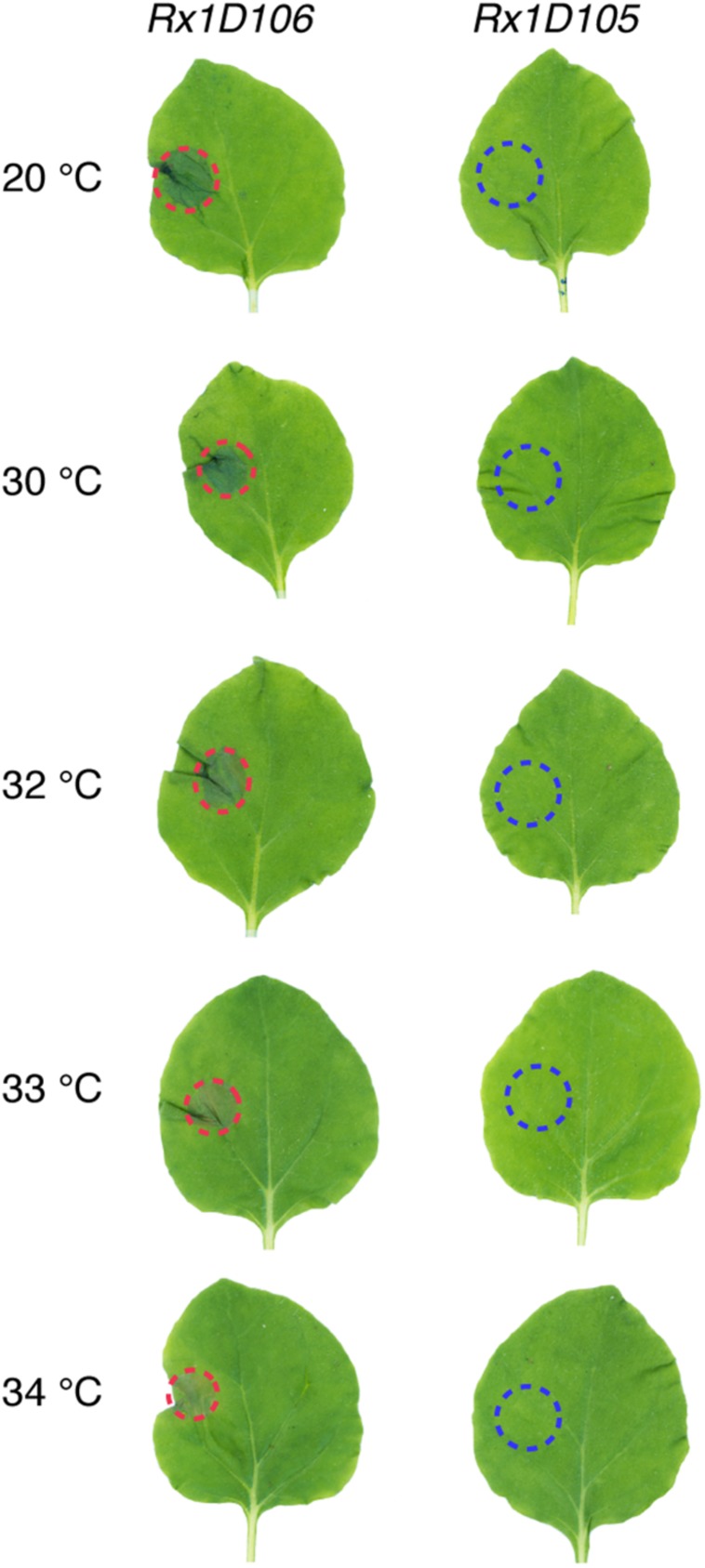
Rx1 triggers HR upon *CP106* expression up to 34°C. Rx1-mediated HR in transgenic lines of *N. benthamiana* expressing both Rx1 and PVX-CP106 (*Rx1D106*) or PVX-CP105 *Rx1D105*) under the control of a dexamethasone-inducible promoter. *CP* expression was induced by spot application of dexamethasone on the left side of the leaf adaxial surface. HR, marked with red dotted lines, was visible in *Rx1D106* lines at all temperatures tested while no cell death was observed in the *Rx1D105* control.

Since PVX infection is fully abolished above 32°C, an alternative approach was used to monitor Rx1 activity at higher temperatures. Rx1 activation by the Coat Protein 106 variant (CP106) of PVX triggers a Hypersensitive Response in *N. benthamiana* that is visible as a necrotic sector. To express CP106, or CP105 as a negative control, we used the CESSNA system to enable inducible expression upon dexamethasone application (Knip et al., 2019). As *Agrobacterium*-mediated transformation is compromised at 27°C and higher (Dillen et al., 1997) we generated stable transgenic plants expressing *Rx1* in combination with *DEX::CP106* or *DEX::CP105*, referred to as *Rx1D106* and *Rx1D105*, respectively). In the absence of dexamethasone, the generated *Rx1D106* and *Rx1D105* transgenic plants were phenotypically identical to WT plants (data not shown). Upon dexamethasone treatment, expression of *CP105* and *CP106* was observed in *Rx1D105* and *Rx1D106*, respectively, within 2 h following application of dexamethasone (Supplementary Figure 3). As anticipated a clear HR was observed at the DEX treated sector in *Rx1D106* but not in *Rx1D105* leaves, as the latter does not trigger Rx1-mediated signaling (Figure 3). Using these hence validated transgenic lines, the capacity of Rx1 to trigger HR in response to CP106 at elevated temperature was examined. Dexamethasone was locally applied on the left side of one leaf of *Rx1D106* and *Rx1D105* plants placed at 20, 30, 32, 33, and 34°C. As shown in Figure 3, Rx1 triggered HR at all temperatures tested, but only upon dexamethasone mediated induction of *CP106*-, but not *CP105-*expression. These results show that the ability of Rx1 to trigger HR upon CP106 perception is not compromised at temperatures up to 34°C.

## Discussion

In this study, we show that Rx1-mediated resistance to PVX::GFP in *N. benthamiana* remains functional up to a temperature at which the virus was no longer infectious. We also show that the non-permissive temperatures for PVX::GFP infection differ between potato and *N. benthamiana* plants. Indeed, PVX::GFP could spread systemically up to 32°C in *N. benthamiana*, while at 30°C PVX::GFP multiplication in inoculated potato leaves was compromised (Supplementary Figure 1) and no systemically spreading virus could be detected in petioles of inoculated leaves (Supplementary Figure 2). In addition, Rx1-triggered HR was also observed at high temperatures (34°C) in *N. benthamiana*. This finding contrasts a previous study detailing that Rx1-mediated HR was abolished above 28°C (Wang et al., 2009). The main difference between both studies is the use of *A. tumefaciens* to express *R* and *Avr* constructs. In our study both genes are stably integrated in the plant genome and *Avr* expression is triggered by applying dexamethasone. In our system co-expressing *Rx1* with the non-recognized CP105 variant did not trigger cell death, thereby ruling out the possibility that dexamethasone itself triggered cell death at elevated temperatures. Considering the fact that *Agrobacterium*-mediated transformation efficiency strongly decreases at increased temperatures, and was shown to be fully compromised at 29°C (Dillen et al., 1997), we propose that the absence of HR observed in Wang et al. (2009) could be attributed to a lack or a poor expression of the construct(s) used.

The decrease of PVX titer in local, and especially in systemic leaves, with increasing temperature has been previously reported in Nicotiana species (Ma et al., 2016). Furthermore, it is likely that PVX::GFP will have a slightly altered performance as compared to a natural strain. We observed that PVX::GFP infectiousness in potato was impaired at lower temperatures than in *N. benthamiana*. *N. benthamiana* is hypersusceptible to many RNA viruses due to a mutation in an RNA dependent RNA Polymerase that is important for antiviral defense based on RNA silencing (Yang et al., 2004). PVX replicates slower in potato than in *N. benthamiana*, which could explain the higher viral titers in *N. benthamiana* as compared to potato at 20°C (Supplementary Figure 1). At elevated temperature (e.g., 30°C) PVX::GFP was not detectable in potato 1 week after inoculation with the virus (Supplementary Figures 1, 2). We cannot exclude whether viral replication and spread is compromised in potato at the elevated temperature, but we could not prolong the incubation time as the plants started to collapse after 1 week. The mechanism underlying poor viral replication and spread is unknown, but could be related to the observation that the RNA silencing machinery in plants is more active at elevated temperatures (Szittya et al., 2003). Further study is required to resolve whether RNA silencing is responsible for impaired viral replication at elevated temperatures in potato.

NRCs are required for resistance mediated by both sensor CNLs and RLPs in Solanaceae (Gabriels et al., 2007; Wu et al., 2017). Several of these CNL- or RLP-mediated resistances are compromised at elevated temperatures (De Jong et al., 2002; Romero et al., 2002; De Carvalho et al., 2015), pointing to NRCs as potential suspects for thermosensitivity. However, our findings show that Rx1-mediated resistance against PVX::GFP is still efficient up to a temperature at which the virus is not infectious anymore. As Rx1 immunity is unaltered at elevated temperature, this means that Rx1 activation and its downstream signaling component are also functional. Rx1 has been shown to require NRC2, 3 or 4 downstream of its activation to trigger HR (Wu et al., 2017). Therefore, it is tempting to speculate that at least one of the Rx1-interacting NRCs functions at elevated temperature. Consequently, the immune sensors (sensor NLRs) are the most probable components that are affected in thermosensitive immune signaling Notably, a similar observation has been made for two TNLs, SNC1 from Arabidopsis and N from tobacco (Zhu et al., 2010). Through genetic screens and targeted mutagenesis, it was shown that SNC1 and N are the thermosensitive components in immune signaling. Together these observations suggest that for both CNL-, TNL-, or RLP- triggered immunity, the sensor is the temperature sensitive element. How a sensor NLR is affected by elevated temperature and loses its activity is unknown. Of note, several studies show that an elevated ambient temperature reduces accumulation of sensor NLR proteins, such as SNC1 or RPS4 and N in the nucleus (Wang et al., 2009; Zhu et al., 2010; Mang et al., 2012; Hua, 2013). For some NLRs a nuclear location has been shown to be crucial for triggering immunity, providing a potential mechanism (Qi and Innes, 2013).

The premise of these observations, that the sensors are thermosensitive while downstream immune signaling components do function at elevated temperatures, is relevant as it provides leads on how to improve thermotolerance of disease resistances by identifying the immune receptor itself as main target for mutagenesis.

## Author Contributions

MR and FT designed the study and wrote the manuscript. MR, MK, TA, and MB performed experiments. MR analyzed the data and drafted all figures. All authors read and approved the final manuscript.

## Conflict of Interest

The authors declare that the research was conducted in the absence of any commercial or financial relationships that could be construed as a potential conflict of interest.

## Footnotes

1 https://www.addgene.org/112941/
