# Supplementary material for: Unlike Many Disease Resistances, Rx1-Mediated Immunity to Potato Virus X Is Not Compromised at Elevated Temperatures
Source: Front Genet. 2020 Apr 24;11:417. doi: 10.3389/fgene.2020.00417 (PMC7193704; doi:10.3389/fgene.2020.00417)
Supplement: Supplementary file 1 [file Data_Sheet_1.PDF]

Supplementary Material

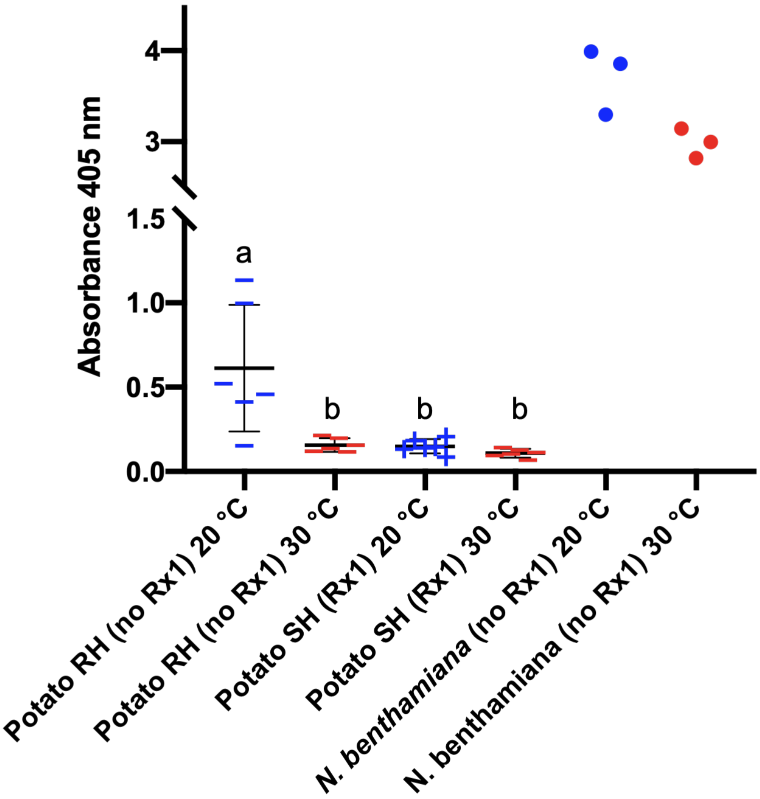

Supplementary Figure 1. Unlike in *N. benthamiana* PVX::GFP is not infectious at 30 °C in potato. PVX quantification was performed by ELISA on rub-inoculated potato or *N. benthamiana* leaves 1-week-post inoculation. Different dilutions of leaf protein extracts were analyzed by ELISA to (from 1 to 1000 times diluted) to quantify viral titers. The results of the 10 times diluted samples are depicted here. At this dilution, the ELISA was quantitative and the biggest difference in virus titer was observed between resistant (SH) and susceptible (RH) potato plants at 20 °C. For potatoes, the average signal and standard deviation (SD) from 6 independently inoculated leaflets are depicted. The means were statistically analyzed with an ANOVA test followed by a post-hoc Tukey HSD multiple comparison and grouped according to significant differences with adjusted p-values < 0.005.

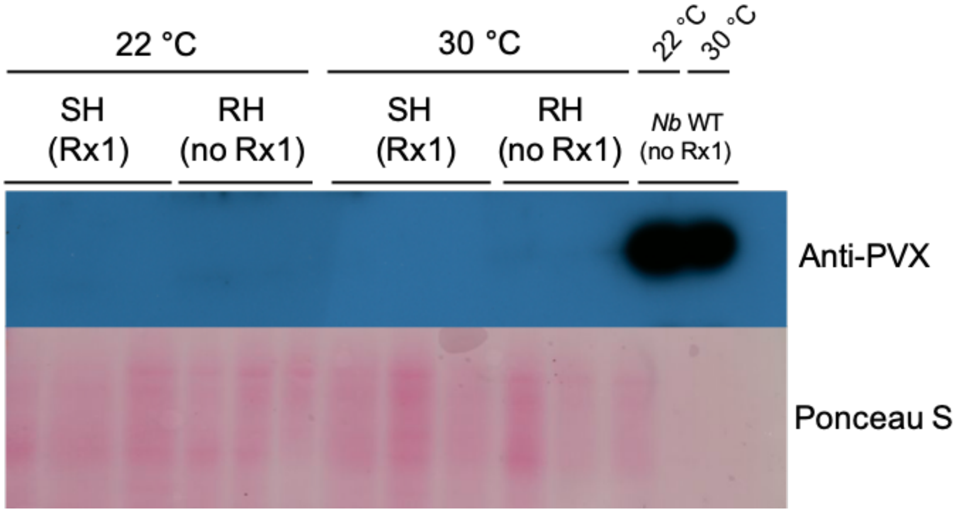

Supplementary Figure 2. PVX::GFP is present in WT *N. benthamiana* but not in potato inoculated leaf petioles one week post inoculation. PVX::GFP detection was performed by western blot using anti-PVX antibodies on the petioles of inoculated leaves.

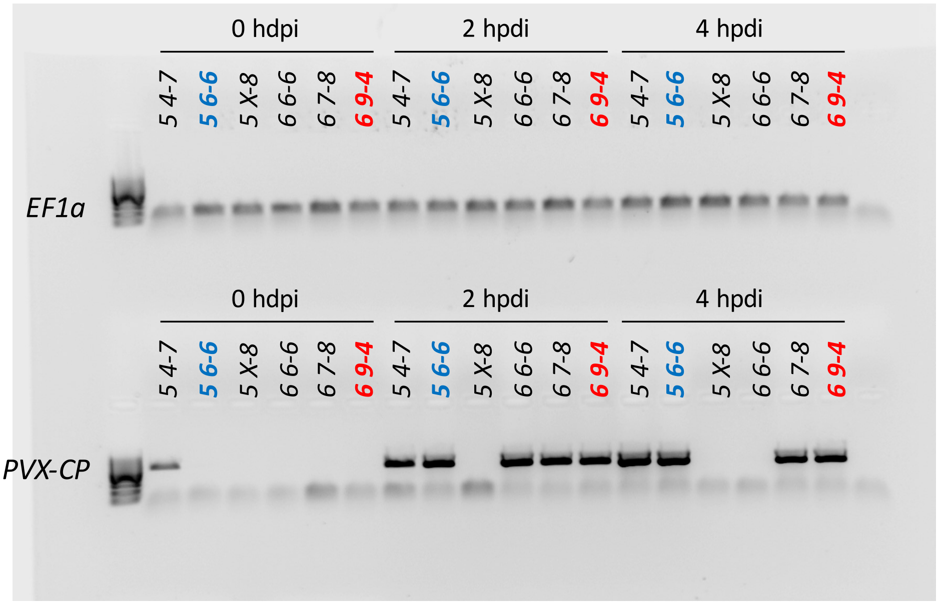

Supplementary Figure 3. CP transcripts detected by RT-PCR in *Rx1D105* (line *5 4-7*, *5 6-6* and *5 X-8*) and *Rx1D106* (line *6 6-6*, *6 7-8* and *6 9-4*) plants generated in this study, 0, 2 and 4 hours post dexamethasone induction (hpdi). Amplification products of *PVX-CP* transcripts, or plant *EF1α* transcripts serving as an internal reference, are shown in the bottom- and top-row, respectively. The transgenic lines used in this study, *Rx1D105 6-6* and *Rx1D106 9-4* are in indicated in blue and red, respectively.
